# Differences in Genotype, Clinical Features, and Inflammatory Potential *of Borrelia burgdorferi* sensu stricto Strains from Europe and the United States

**DOI:** 10.3201/eid2205.151806

**Published:** 2016-05

**Authors:** Tjasa Cerar, Franc Strle, Dasa Stupica, Eva Ruzic-Sabljic, Gail McHugh, Allen C. Steere, Klemen Strle

**Affiliations:** University of Ljubljana, Ljubljana, Slovenia (T. Cerar, E. Ruzic-Sabljic);; University Medical Center, Ljubljana (F. Strle, D. Stupica);; Massachusetts General Hospital, Boston, Massachusetts, USA (G. McHugh, A.S. Steere, K. Strle)

**Keywords:** Lyme borreliosis, Borrelia burgdorferi sensu stricto, bacteria, B. afzelii, B. garinii, genotype, RNA intergenic spacer type, outer surface protein C, multilocus sequencing typing, erythema migrans, clinical features, inflammatory potential, Slovenia, Europe, United States

## Abstract

*Borrelia burgdorferi* sensu stricto isolates from patients with erythema migrans in Europe and the United States were compared by genotype, clinical features of infection, and inflammatory potential. Analysis of outer surface protein C and multilocus sequence typing showed that strains from these 2 regions represent distinct genotypes. Clinical features of infection with *B. burgdorferi* in Slovenia were similar to infection with *B. afzelii* or *B. garinii*, the other 2 *Borrelia* spp. that cause disease in Europe, whereas *B. burgdorferi* strains from the United States were associated with more severe disease. Moreover, *B. burgdorferi* strains from the United States induced peripheral blood mononuclear cells to secrete higher levels of cytokines and chemokines associated with innate and Th1-adaptive immune responses, whereas strains from Europe induced greater Th17-associated responses. Thus, strains of the same *B. burgdorferi* species from Europe and the United States represent distinct clonal lineages that vary in virulence and inflammatory potential.

## Abstract 2

Strains from the United States are more virulent and have greater inflammatory potential.

Incidence of Lyme borreliosis, the most common vectorborne disease in the Northern Hemisphere, is increasing. This disease usually begins with an expanding skin lesion, erythema migrans, which is often accompanied by nonspecific symptoms, such as headache, fatigue, myalgias, and arthralgias. If not treated, this infection can disseminate to the nervous system, heart, or joints (*1**,**2*).

Lyme borreliosis is caused primarily by 3 species of the *Borrelia burgdorferi* sensu lato complex: *B. afzelii*, *B. garinii*, and *B. burgdorferi* sensu stricto (hereafter referred to as *B.* *burgdorferi*) (*3*). Variations in geographic distribution and clinical manifestations of this disease have been noted for each species. In Europe, infection is predominantly with *B. afzelii,* which usually remains localized to the skin, and *B. garinii*, which is usually associated with nervous system involvement (*1*). *B. burgdorferi* infection is rare in Europe; little is known about its clinical course there. In the United States, *B. burgdorferi* is the sole agent of Lyme borreliosis; in the northeastern United States, it is particularly arthritogenic (*1**,**2*). For all 3 species, the first sign of infection is often an erythema migrans lesion. However, *B. burgdorferi* infection in the United States is associated with a greater number of disease-associated symptoms and more frequent hematogenous dissemination than *B. afzelii* or *B. garinii* infection in Europe (*4**–**7*).

Clinical manifestations of Lyme borreliosis are believed to result from host immune response to the spirochete. Erythema migrans lesions of *B. burgdorferi*–infected patients in the United States have higher levels of mRNA for cytokines and chemokines associated with innate and Th1-adaptive immune responses than lesions from patients in Austria infected with *B. afzelii* (*4*). *B. burgdorferi*–infected patients in the United States also have higher levels of cytokines and chemokines in serum, and isolates from these patients induce macrophages to secrete more interleukin-6 (IL-6), IL-8, IL-10, chemokine ligand 3 (CCL3), CCL4, and tumor necrosis factor (TNF) than *B. afzelii* or *B. garinii* from patients in Slovenia (*8*).

These differences in inflammatory potential between *B. burgdorferi* from the United States and *B. afzelii* and *B. garinii* from Europe might account, in part, for differences in clinical manifestations of Lyme borreliosis. However, little is known about virulence and inflammatory capacity of *B. burgdorferi* in Europe, or how it compares with *B. burgdorferi* in the United States. In this study, we compared infection with *B. burgdorferi* in Europe and the United States by genotype, clinical manifestations, and inflammatory potential.

## Methods

### Patients and Strains

Twenty-nine *B. burgdorferi* isolates were cultured from Lyme borreliosis patients in Slovenia (Central Europe) at the Institute of Microbiology and Immunology (Ljubljana, Slovenia). Isolates were identified as *B. burgdorferi* by using *Mlu*I large restriction fragment patterns (*9*). Twenty-four isolates were obtained from skin (19 from erythema migrans, 4 from acrodermatitis chronica atrophicans, 1 from borrelial lymphocytoma), and 5 from cerebrospinal fluid of patients with Lyme neuroborreliosis. These 29 strains represent all available patient-derived *B. burgdorferi* isolates collected during a 20-year period (1994–2013) at the Institute of Microbiology and Immunology. Clinical information and demographic data were obtained for same 29 patients at the Lyme Borreliosis Outpatient Clinic at the University Medical Center Ljubljana. The study was approved by Slovenian National Medical Ethics Committee (no.133/06/13).

In a study of patients with Lyme borreliosis in the United States (Rhode Island and Connecticut), 91 *B. burgdorferi* isolates were cultured from erythema migrans lesions (*10*). One isolate had a mixed genotype and was not included in our study. All patients met the Centers for Disease Control and Prevention (Atlanta, GA, USA) criteria for erythema migrans. The Human Investigation Committee at Tufts Medical Center and Massachusetts General Hospital approved this study.

### Characterization of Strains

We genotyped *Borrelia* strains by using outer surface protein C (OspC), ribosomal RNA intergenic spacer type (RST), and multilocus sequence typing (MLST). OspC type was determined by using seminested PCR and sequencing. RST was determined by using nested PCR and restriction fragment length polymorphism analysis (*11**,**12*). OspC and RST genotypes were determined for all 29 *B. burgdorferi* isolates from Slovenia and 90 from the United States.

For MLST analysis, we amplified 8 chromosomal housekeeping genes (*clpA*, *clpX*, *nifS*, *pepX*, *pyrG*, *recG*, *rplB*, and *uvrA*) by using nested PCR, sequenced in both directions (*13*), and analyzed by using CLC Main Workbench (http://www.clcbio.com/products/clc-main-workbench/). MLST analysis included 29 isolates from Slovenia and a representative subset of 41 isolates comprising the most common *B. burgdorferi* subtypes from the northeastern United States. Because of the prevalence of OspC type B among isolates from Slovenia, this analysis also included all 11 OspC type B isolates from the United States in our collection. New sequence types were submitted to the MLST database (http://pubmlst.org/borrelia/).

### Phylogenetic Analysis

We constructed a minimum spanning tree by using BioNumerics version 7.1 software (Applied Maths, Austin, TX, USA). Comparison of strains from different regions included MLSTs of human *B. burgdorferi* isolates in the *Borrelia* MLST database. Phylogenetic trees of concatenated sequences of housekeeping genes were constructed by using MrBayes software (*14*).

### Comparison of Clinical Findings

Demographic and clinical findings were available for 14 of 19 erythema migrans patients with *B. burgdorferi* infection from Slovenia and 90 erythema migrans patients with *B. burgdorferi* infection from the northeastern United States. Findings for *B. burgdorferi* infection we compared findings for 200 patients in Slovenia with *B. afzelii* infection and 116 with *B. garinii* infection; all had culture-positive erythema migrans.

### Inflammatory Potential of *B. burgdorferi* Isolates

We assessed the inflammatory capacity of *B. burgdorferi* strains by stimulating peripheral blood mononuclear cells (PBMC) with 29 *B. burgdorferi* isolates and determining levels of cytokines and chemokines in culture supernatants. These isolates included 14 *B. burgdorferi* isolates from erythema migrans lesions of patients from Slovenia for whom detailed clinical information was available and 15 representative *B. burgdorferi* isolates (5 each of RST1, RST2, and RST3) from patients in the United States. For cell culture experiments, 29 low-passage (<5) isolates were grown to mid-to-late log phase in complete Barbour-Stoenner-Kelly medium (Sigma-Aldrich, St. Louis, MO, USA) (*15*). Numbers of spirochetes in each culture were determined by optical density using a standard curve (*8*).

Human PBMC were obtained from 4 healthy donors at the Massachusetts General Hospital Blood-Component Laboratory, and PBMC were isolated from leukopaks by centrifugation in lymphocyte separation medium (MP Biomedicals, Santa Ana, CA, USA). Cells were cultured overnight in RPMI 1640 medium containing 10% human serum in 96-well plates (2 × 10^5^ cells/well) at 37°C in 5% CO_2_. To keep host factors constant, we stimulated PBMC from each healthy donor with each of 29 patient-derived *B. burgdorferi* isolates (multiplicity of infection = 25) in 4 independent experiments for 7 days (*16*).

We assessed protein levels of 22 cytokines and chemokines associated with innate and adaptive immune responses (innate: TNF, IL-1β, IL-6, IL-10, granulocyte–macrophage colony-stimulating factor, IL-8, IFN-α, CCL2, CCL3, and CCL19; adaptive-Th1: IFN-γ, IL-12p40, IL-12p70, cysteine-X-cysteine motif cytokine ligand 9 (CXCL9), and CXCL10; adaptive-Th17: IL-17A, IL-17F, IL-21, IL-22, IL-23, IL-25, and IL-27) in culture supernatants by using bead-based Luminex (EMD-Millipore, Darmstadt, Germany) multiplex assays. We averaged results from 4 experiments for analysis.

### Statistical Analysis

We assessed differences between groups by using the Mann-Whitney rank-sum test and differences for categorical data by using the Fisher exact test (SigmaPlot-12.5; http://www.sigmaplot.com/products/sigmaplot/produpdates/prod-updates18.php0). p values <0.05 were considered significant.

## Results

### *B. burgdorferi* Genotypes

**Figure 1:**
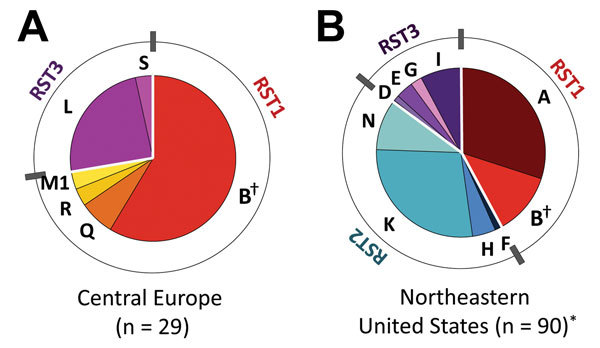
Distribution of *Borrelia burgdorferi* sensu stricto strains by outer surface protein C (OspC) and ribosomal RNA intergenic spacer type (RST). A) 29 isolates from patients with Lyme borreliosis in cental Europe (Slovenia). B) 90 isolates from patients with erythema migrans in the northeastern United States. OspC types are indicated by letters, and RSTs are indicated by colors. Red, RST1; blue, RST2; purple, RST3. *Based on previously published data (*10*). †Denotes OspC genotype (OspC type B) found in central Europe and the United States.

To examine genotypic characteristics of *B. burgdorferi* from Europe and the United States, we assessed 29 patient-derived isolates from Slovenia and 90 isolates from the United States by using the 2 most common typing systems, RST and OspC, which show strong linkage disequilibrium. Of 29 isolates from Slovenia, 21 (72%) were RST1 and 8 (28%) were RST3; none were RST2 (Figure 1; Table 1). Of 90 isolates from the United States, 38 (42%) were RST1, 39 (43%) were RST2, and 13 (14%) were RST3, a distribution consistent with those of other studies (*6**,**10**,**17**,**18*).

**Table 1:** Characterization of *Borrelia burgdorferi* sensu stricto strains, by RST and OspC, from patients in Slovenia and the United States*

Genotype	Slovenia, n = 29, no. (%)	United States, n = 90†, no. (%)
RST1	21 (72)	38 (42)
OspC type A	0	27 (30)
OspC type B‡	17 (58)	11 (12)
OspC type Q	2 (7)	0
OspC type R	1 (3)	0
OspC type M1	1 (3)	0
RST2	0	39 (43)
OspC type F	0	1 (1)
OspC type H	0	4 (4)
OspC type K	0	25 (28)
OspC type N	0	9 (10)
RST3	8 (28)	13 (14)
OspC type D	0	1 (1)
OspC type E	0	3 (3)
OspC type G	0	2 (2)
OspC type I	0	7 (8)
OspC type L	7 (24)	0
OspC type S	1 (3)	0

*RST, ribosomal RNA intergenic sequence type; OspC, outer surface protein C. †Based on previously published data (*10*). ‡Found in central Europe and the United States.

OspC typing showed that OspC type B (RST1) was the only OspC type found among isolates from Europe and the United States. Other OspC types were found exclusively in Slovenia (Q, R, L, S) or the United States (A, F, K, N, D, E, G, I). The most common *B. burgdorferi* strains in Slovenia were RST1-OspC type B (58%) and RST3-OspC type L (24%), whereas the most common strains in the northeastern United States were RST1-OspC type A (30%) and RST2-OspC type K (28%).

MLST analysis (Table 2) included 29 isolates from Slovenia and 41 isolates from the United States, which comprised the major OspC types (A, K, and I) in the northeastern United States and all 11 OspC type B strains in our collection. Analysis identified 15 sequence types (STs). There was no overlap in STs from Europe and the United States, which demonstrated that strains of the same *Borrelia* species, including OspC type B strains, from the 2 regions were distinct genotypes.

**Table 2:** Characterization of *Borrelia burgdorferi* sensu stricto strains, by MLST genotyping, from patients in Slovenia and United States*

Genotype	No. (%) patients	
	Slovenia, n = 29	United States, n = 41†
RST1	21	21
OspC type A	0	10
MLST ST1	0	10 (24)
OspC type B	17	11
MLST ST20	6 (20)	0
MLST ST314	6 (20)	0
MLST ST545‡	5 (17)	0
MLST ST59	0	10 (24)
MLST ST7	0	1 (2)
OspC type Q	2	0
MLST ST20	1 (3)	0
MLST ST546‡	1 (3)	0
OspC type R	1	0
MLST ST20	1 (3)	0
OspC type M1	1	0
MLST ST20	1 (3)	0
RST2	0	10
OspC type F	0	1
MLST ST8	0	1 (2)
OspC type K	0	7
MLST ST3	0	6 (15)
MLST ST9	0	1 (2)
OspC type N	0	2
MLST ST9	0	2 (5)
RST3	8	10
OspC type D	0	1
MLST ST38	0	1 (2)
OspC type E	0	2
MLST ST19	0	2 (5)
OspC type G	0	2
MLST ST14	0	2 (5)
OspC type I	0	5
MLST ST16	0	5 (12)
OspC type L	7	0
MLST ST24	7 (24)	0
OspC type S	1	0
MLST ST24	1 (3)	0

*MLST, multilocus sequence typing; RST, ribosomal RNA intergenic sequence type; OspC, outer surface protein C; ST, sequence type. †The 41 isolates from the United States were selected from a larger cohort that were representative of the most common RST/OspC subtypes in the northeastern United States. These isolates included all available OspC type B strains in our collection. ‡New ST.

Isolates from Slovenia represented 3 previously described STs and 2 new STs (ST545 and ST546) not in the MLST database. Strains from the United States represented a more heterogeneous group of 10 STs, all of which were reported previously in the MLST database. Three STs (ST9, ST20, and ST24) each comprised >1 OspC type. This result might be explained by greater propensity for horizontal transfer of genetic information at *ospC* gene loci, which are apparent recombination hotspots (*19*), whereas the 8 loci used in MLST analysis have lower rates of genetic recombination.

**Figure 2:**
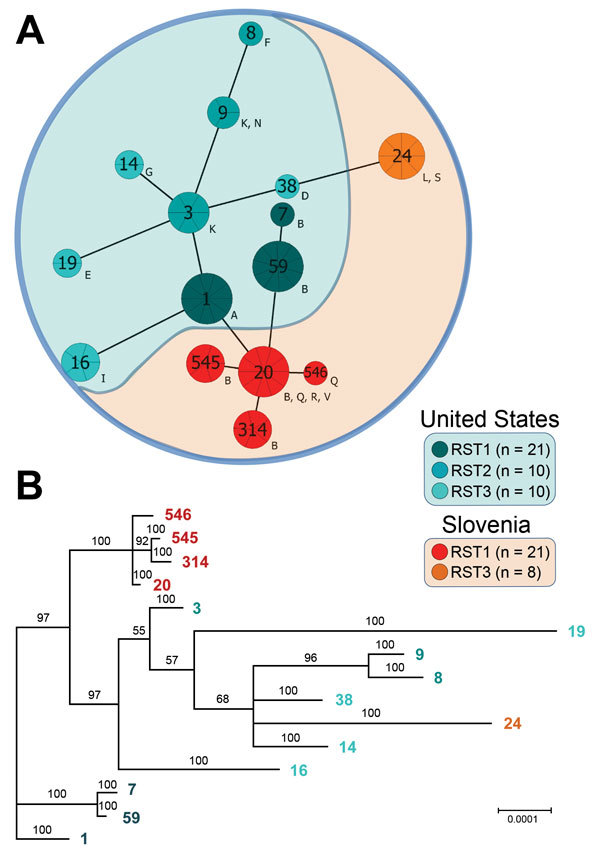
Phylogenetic analysis of *Borrelia burgdorferi* sensu stricto strains from central Europe (Slovenia) and the United States. A) Minimum spanning tree analysis of 70 isolates included in this study. Sequence types (STs) are indicated by numbers, and outer surface protein types are indicated by letters. Sizes of circles indicate ST sample sizes. Lengths of lines connecting STs indicate extent of variation (order of certainty) (no. locus variants). STs connected by the shortest black line are single-locus variants. Letters outside circles indicate OspC types. RST, ribosomal RNA intergenic spacer. B) Bayesian consensus tree resulting from simultaneous analysis of concatenated sequences of 8 housekeeping genes (*clpA*, *clpX*, *nifS*, *pepX*, *pyrG*, *recG*, *rplB*, and *uvrA*). Values at nodes indicate Bayesian posterior probabilities (proportion of sampled trees containing the taxon bipartition). Scale bar indicates nucleotide substitutions per site.

Minimum spanning tree and phylogenetic analyses of 70 *B. burgdorferi* strains underscored differences between strains in the 2 regions (Figure 2). Strains from Europe formed 2 clonal complexes (CC20 and CC24), which were separated by 3 and 6 alleles, respectively, from most the closely related strains in the United States.

**Figure 3:**
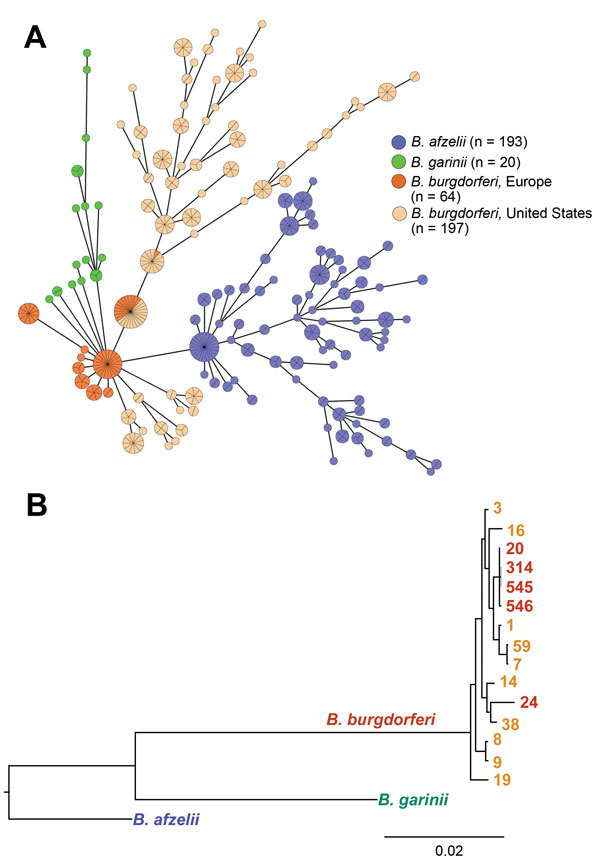
Phylogenetic comparison of 3 major pathogenic *Borrelia* species (*Borrelia afzelii*, *B. garinii*, and *B. burgdorferi* sensu stricto) that cause Lyme borreliosis. A) Minimum spanning tree analysis of 474 *B. burgdorferi* sensu lato human isolates. Analysis included 404 previously published datasets available in the multilocus sequence typing database (http://pubmlst.org/borrelia/) as of May 5, 2015, and 70 *B. burgdorferi* sensu stricto isolates from this study. Circles and numbers indicate specific sequence types (STs). Sizes of circles indicate MLST sample size and colors indicate origin of isolates. Lengths of lines connecting STs indicate order of certainty. STs connected by the shortest line are single locus-variants. B) Bayesian consensus tree resulting from simultaneous analysis of concatenated sequences of housekeeping genes of 70 *B. burgdorferi* sensu stricto isolates included in this study, representative strains of *B. afzelii* (http://pubmlst.org/borrelia/id:1546), and *B. garinii* (http://pubmlst.org/ borrelia/id:1829). Values at nodes indicate Bayesian posterior probabilities (proportion of sampled trees containing the taxon bipartition). Scale bar indicates nucleotide substitutions per site.

Comparison of *B. burgdorferi* with *B. afzelii* or *B. garinii* (Figure 3) showed deeper branching among different *Borrelia* species but shallower branching within the same species, which demonstrated greater genetic discordance among species. Nevertheless, genotypic differences between *B. burgdorferi* in Europe and the United States, and closer clustering of strains within each geographic region, suggest divergence of *B. burgdorferi* on the 2 continents.

### Clinical Findings for Patients with Erythema Migrans

We compared 14 *B. burgdorferi*–infected patients in Slovenia for whom detailed clinical information was available with 90 patients in the United States and found that patients from the United States had significantly shorter duration of erythema migrans at diagnosis (4 vs. 7 days; p = 0.02), greater frequency of associated symptoms (78% vs. 29%; p<0.001), and greater number of associated symptoms (4 vs. 0 symptoms; p = 0.005) (Table 3). *B. burgdorferi* from the United States differed from *B. afzelii* and *B. garinii* for most clinical parameters measured. In contrast, clinical features of *B. burgdorferi* infection in Slovenia were similar to those for *B. afzelii* or *B. garinii* infections (Table 3), despite substantial genotypic differences among species (Figure 3).

**Table 3:** Clinical characteristics of erythema migrans patients infected with *Borrelia afzelii*, *B. garinii*, or *B. burgdorferi* sensu stricto in Slovenia and *B. burgdorferi* sensu stricto in the United States*

Characteristic	Slovenia	United States, *B. burgdorferi *sensu stricto, n = 90†	p value‡

*B. afzelii*, n = 200†	*B. garinii*, n = 116†	*B. burgdorferi *sensu stricto, n = 14
General					
Age, y	50 (15–79)	54 (20–83)	50 (3–77)	49 (15–82)	0.6
Female sex	128 (64)	61 (53)	10 (71)	44 (49)	0.2
EM duration at study entry, d	9 (1–206)	5 (1–61)	7 (2–53)	4 (1–30)	**0.02**
EM diameter, cm	13 (5–86)	18 (5–72)	12 (5–38)	11 (5–56)	0.6
Symptoms at study entry					
Symptoms/patient	0 (0–6)	0 (0–7)	0 (0–6)	4 (0–9)	**0.005**
Patients with symptoms	66 (33)	43 (37)	4 (29)	70 (78)	**<0.001**
Fatigue	51 (26)	23 (20)	3 (21)	55 (61)	**0.01**
Arthralgia	30 (15)	21 (18)	3 (21)	42 (47)	0.1
Myalgia	28 (14)	22 (19)	3 (21)	28 (32)	0.7
Headache	27 (14)	21 (18)	4 (29)	45 (50)	0.2
Fever, chills	12 (6)	10 (9)	2 (14)	41 (46)	**0.05**
Neck stiffness	9 (5)	4 (3)	0	43 (48)	**0.002**
Malaise	21 (11)	21 (18)	2 (14)	47 (52)	**0.01**

*Values are median (range) or no. (%). EM, erythema migrans.  †Based on previously published data: *B. burgdorferi sensu stricto *(*10*); *B. afzelii *(*5*); *B. garinii *(*20*). ‡For comparison of *B. burgdorferi* sensu stricto from Slovenia versus the United States. Values in bold indicate statistical significance.

**Figure 4:**
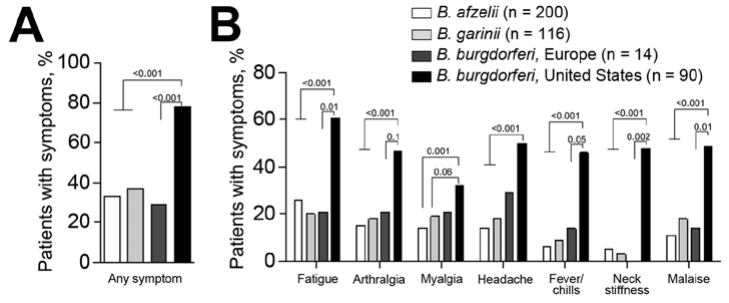
Frequency of symptoms in patients with erythema migrans infected with *Borrelia afzelii*, *B. garinii*, or *B. burgdorferi* sensu stricto from central Europe and *B. burgdorferi* sensu stricto from the United States. A) Any symptom, B) individual symptoms. Patients were assessed for 8 symptoms (fatigue, arthralgia, myalgia, headache, fever, chills, neck stiffness, or malaise). White bars indicate patients from Europe infected with *B. afzelii*, light grays bars indicate patients from Europe infected with *B. garinii*, dark gray bars indicate patients from Europe infected with *B. burgdorferi* sensu stricto, and black bars indicate patients from the United States infected with *B. burgdorferi* sensu stricto. Differences between strains were assessed by using the Fisher exact test. p values are indicated. There were no differences between *B. afzelii*, *B. garinii*, or *B. burgdorferi* sensu stricto from Slovenia.

Differences between *Borrelia* species from Europe and the United States were most apparent for symptomology of infection (Figure 4). In the United States, erythema migrans is typically associated with fever, neck stiffness, malaise, and fatigue. However, these symptoms were substantially less common in Slovenia. Thus, despite greater phylogenetic similarity with *B. burgdorferi* from the United States, infection with *B. burgdorferi* strains from Europe reflected more closely findings for 2 genetically discordant *Borrelia* species in Europe (*B. afzelii* and *B. garinii*) with which it shares an ecologic niche. In contrast, *B. burgdorferi* from the United States appeared to be more virulent.

### Inflammatory Responses in PBMC Stimulated with *B. burgdorferi*

To determine whether *B. burgdorferi* from the United States and Europe vary in inflammatory potential, we assessed levels of 22 cytokines and chemokines in supernatants of healthy human PBMC stimulated with 14 *B. burgdorferi* isolates from patients in Slovenia and 15 representative isolates from patients in the United States (5 each RST1, RST2, and RST3), for whom detailed clinical information was available. All 29 isolates were tested by using PBMC from each of 4 healthy donors, and results from 4 experiments were averaged for analysis.

**Figure 5:**
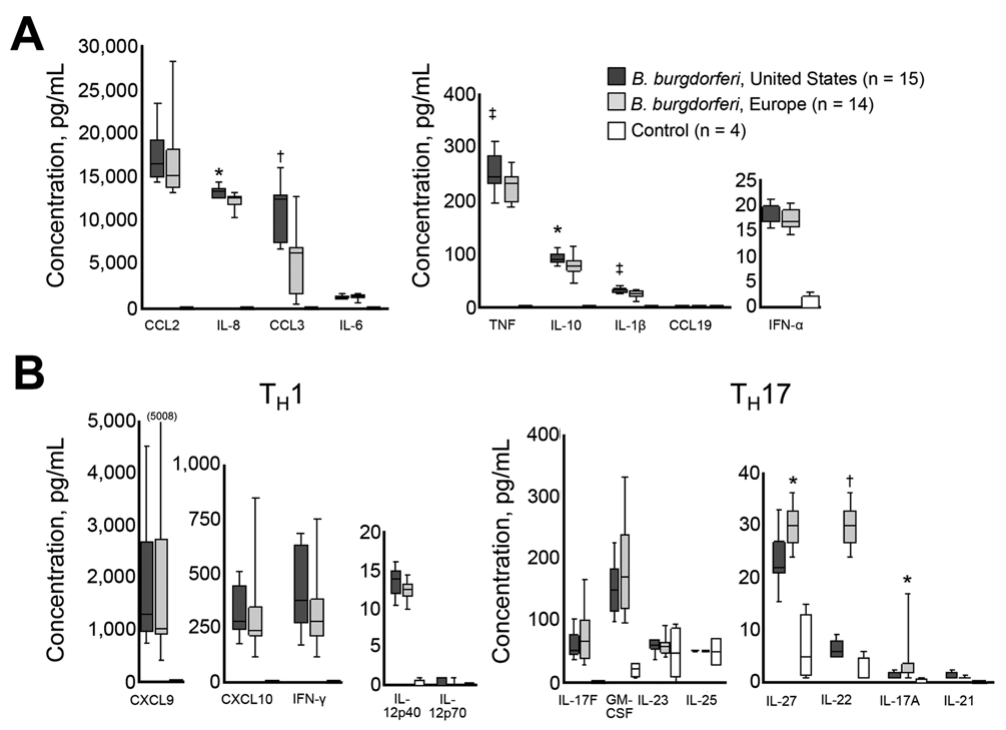
Inflammatory potential of *Borrelia burgdorferi* sensu stricto from Europe and the United States. Levels of 22 cytokines and chemokines associated with innate (A) or adaptive (B) immune responses. Innate responses: tumor necrosis factor (TNF), interleukin-1β (IL-1β), IL-6, IL-10, granulocyte–macrophage colony-stimulating factor (GM-CSF), IL-8, chemokine ligand 2 (CCL2), CCL3, and CCL19. Adaptive immune responses: Th1: interferon-γ (IFN-γ), IFN-α, IL-12p40, IL-12p70, cysteine-X-cysteine motif chemokine ligand 9 (CXCL9), and CXCL10; Th17: IL-17A, IL-17F, IL-21, IL-22, IL-23, IL-25, and IL-27. Immune responses were assessed in peripheral blood mononuclear cell culture supernatants after 7 days of stimulation with 14 *B. burgdorferi* sensu stricto isolates from Europe or 15 *B. burgdorferi* sensu stricto isolates from the United States (multiplicity of infection = 25) by using bead-based Luminex (EMD-Millipore, Darmstadt, Germany) multiplex assays. Each of 29 *B. burgdorferi* sensu stricto isolates was tested with peripheral blood mononuclear cells from each of 4 healthy donors in 4 independent experiments. Cytokine and chemokine values from the 4 experiments were averaged for analysis. Data are presented as box and whisker plots, boxes indicate interquartile ranges (1st and 3rd quartiles), lines inside boxes indicate median values, and error bars indicate 10th and 90th percentiles (value in parenthesis indicates the highest value). For comparison of *B. burgdorferi* sensu stricto isolates from Slovenia or the United States, *p<0.01, †p<0.001, ‡p<0.05.

Isolates from the United States and Europe induced greater expression of most cytokines and chemokines tested compared with unstimulated controls (Figure 5). However, *B. burgdorferi* from the United States induced higher levels of several mediators associated with innate immune responses, including IL-1β, IL-8, IL-10, TNF, and CCL3, than did *B. burgdorferi* from Europe (Figure 5, panel A). A similar trend was observed for Th1-associated mediators (IL-12p40, INF-γ, INF-γ–inducible CXCL9, and CXCL10), which are strong chemoattractants for CD4+/CD8+ T-effector cells (Figure 5, panel B). In contrast, levels of several Th17 mediators, including IL-17A, IL-22, and IL-27, were higher in cells stimulated with *B. burgdorferi* from Europe. Because immune response seems to be critical in disease expression, these differences in inflammatory responses might contribute to differences in clinical features of Lyme borreliosis in Europe and the United States.

## Discussion

We compared infection with *B. burgdorferi* in Europe and the United States by genotype, clinical manifestations, and inflammatory potential. Strains in Europe differed from strains in the United States for all 3 parameters, which demonstrates transcontinental diversification of this species.

Although *B. burgdorferi* from Slovenia and the United States are depicted as the same species, they represent distinct clonal complexes that vary in capacity to induce host inflammatory immune responses and clinical features of disease. Clinical and immune characteristics of *B. burgdorferi* from Europe more closely resemble those of the phylogenetically distinct species *B. afzelii* and *B. garinii* from Europe, with which they share an ecological niche, than those of *B. burgdorferi* from the northeastern United States. These findings underscore divergence of *B. burgdorferi* strains on 2 continents. Moreover, data indicate a convergence of certain features among disparate *Borrelia* species within the same region, presumably through sharing of genetic information (*21*).

Three RST and >30 OspC genotypes have been identified in *B. burgdorferi* obtained from various sources (*11**,**12*), including 24 OspC subtypes that cause infection in humans (*22*). On the basis of 3 studies that evaluated genotypes of *B. burgdorferi* isolates that were obtained primarily from ticks from Europe or the United States (*13**,**22**,**23*), most OspC types were believed to be present exclusively in North America or Europe, whereas a few OspC types (A, B, K, E, L) were present in both continents (*22**–**24*).

Our study of *B. burgdorferi* from patients in Slovenia or the United States partially corroborates these findings and demonstrates that strains from these 2 regions are genetically distinct. However, of 15 OspC types identified, OspC type B was the only genotype found among isolates from both Slovenia and the United States. The high frequency of OspC type B (58%) in patients from Slovenia and absence of OspC types A, K, or E, which were reported in several countries in Europe (*13**,**22**,**23*), suggests that there might be regional variation in distribution of *B. burgdorferi* in different locations in Europe. Moreover, although not found in the northeastern United States, the high prevalence of OspC type L among patients in Slovenia and its recovery from patients in the midwestern United States (*24*) indicate that this strain also causes human disease.

Analysis of human of *B. burgdorferi* isolates by MLST confirmed that strains on the 2 continents represent different clonal complexes, which is consistent with previous findings in isolates from ticks (*13**,**25*). STs of the 29 isolates from Slovenia differed from STs of the 157 isolates from North America in the MLST database, which implies transcontinental diversification of *B. burgdorferi*. Moreover, Phylogenetic and minimum spanning tree analyses showed that isolates from Slovenia clustered in 2 distinct groups (ST20 and ST24), which suggests that strains in these groups evolved independently.

Although speculative, the data suggest that there might have been 2 independent divergence events for *B. burgdorferi* in Europe and the United States. One event involved migration of ST20, which is most closely related to RST1-OspC type A and B strains that are prevalent in the northeastern United States. A separate event involved ST24, which is closely related to OspC type L strains that were found in patients from the midwestern United States, but not in patients from the northeastern United States. However, these insights are based on a small sample size of isolates, all from humans. Genome analyses of larger numbers of isolates from various sources could provide better resolution of the geographic spread of *B. burgdorferi* (*19*).

Species determination is based on sequence homology of 16S rRNA, a highly conserved chromosomal region of the *Borrelia* genome that undergoes slow evolutionary change. However, ≈40% of borrelial DNA is located on plasmids, including highly polymorphic genes, such as *ospC* (*26*), which encode immunogenic proteins involved in spirochetal virulence. These genes are believed to be under considerable evolutionary pressure, and because of plasmid plasticity, there is evidence for gene recombination and lateral transfer of genetic information among strains (*21*). Thus, despite comprising the same species on the basis of 16S rRNA, *B. burgdorferi* from Europe and the United States appear divergent in expression of genes, such as *ospC*, that are responsible for immunogenicity and virulence. Consequently, strains defined as the same species might cause a spectrum of disease with different clinical features. Thus, species determination on the basis of genetically conserved regions of the genome might not adequately reflect the difference in virulence.

Researchers have reported differences in clinical features of infection with *B. garinii* and *B. afzelii* in Europe and *B. burgdorferi* in the northeastern United States (*4**,**5**,**20**,**27**–**30*). We extended these findings by showing differences in clinical features of erythema migrans caused by *B. burgdorferi* in Slovenia compared with *B. burgdorferi* in the United States. Despite substantial phylogenetic discordance among species, clinical features of infection with *B. burgdorferi* in Slovenia more closely resembled those of milder infections with *B. afzelii* and *B. garinii*, the 2 other *Borrelia* species that cause disease in Europe, than the more symptomatic infection associated with more closely phylogenetically related *B. burgdorferi* from the United States. These findings suggests sharing of genetic information among different *Borrelia* species.

Although we cannot exclude the possibility that host genetic or cultural differences might contribute to differences in clinical features of Lyme borreliosis in Slovenia and the United States, we do not believe that these differences are major factors. First, all study patients at both sites were of European descent and probably similar genetically. Second, evaluation of patients in both locations was similar and included assessment of objective measures, such as fever and erythema migrans diameter and duration, which would not be influenced by cultural differences in reporting symptoms. Third, median duration of erythema migrans in patients in the United States likely was shorter because these patients had more associated symptoms and sought treatment sooner than patients in Slovenia. In support of this interpretation, patients in Europe with *B. garinii* infection, which causes more pronounced itching and burning of erythema migrans lesions than other *Borrelia* species (*29*), had a similar duration of erythema migrans at study entry as patients in the United States. Thus, we believe that differences among *Borrelia* strains are the critical factor in explaining differences in clinical features of Lyme borreliosis on the 2 continents.

Our analysis in this study focused on clinical features of erythema migrans. However, distinctions in disease pathogenesis between *B. burgdorferi* strains from Slovenia and the United States are probably not limited to early disease. Of 29 *B. burgdorferi* isolates from Slovenia, 1 was obtained from a patient with borrelial lymphocytoma, and 4 were obtained from patients with acrodermatitis chronica atrophicans, a late disease manifestation. These clinical manifestations are rarely, if ever, seen in the northeastern United States, where late in the disease, *B. burgdorferi* is commonly associated with development of arthritis, which occurs rarely in Europe. These observations are consistent with a recent report suggesting higher frequency of Lyme neuroborreliosis after *B. burgdorferi* infection in Europe than in the United States (*25*). Thus, clinical differences between *B. burgdorferi* in Europe and the United States probably involve manifestations other than those associated with early infection.

Although there is a range of inflammatory potential for a given *Borrelia* RST strain (*31*), we previously showed in vivo and in vitro that *B. burgdorferi* from the northeastern United States induced greater inflammatory responses than *B. afzelii* and *B. garinii* from Europe (*4**,**8*). In this study, we demonstrated in cell culture that *B. burgdorferi* from Europe and the United States vary in inflammatory potential. Strains from the United States induced higher levels of cytokines and chemokines associated with innate immune responses and showed a similar trend for Th1-associated mediators. In contrast, strains from Europe induced higher levels of several Th17-associated cytokines.

The functional consequence of these differential immune responses is not known. Th17-associated mediators are detected in only a subset of patients with Lyme borreliosis and might not be as effective in spirochetal killing as innate or Th1-adaptive responses (*32*). However, because Lyme borreliosis patients are given antibiotic drugs, it is not known whether the natural history of the disease would be different in patients with predominantly Th1 or Th17 responses to the spirochete.

In addition to divergence of *B. burgdorferi* between Europe and the United States, emerging evidence suggests regional strain variation on each continent. A study of *B. burgdorferi* from humans in the midwestern and northeastern United States reported differences among isolates at these locations (*24*). In the northeastern United States, OspC types A (≈30%) and K (≈30%) are most common, whereas there is greater diversity in the midwestern United States, and OspC type H (≈20%) is most common. Although not assessed systematically, we believe that Lyme borreliosis is a generally milder disease in the midwestern United States. Similarly, the predominance of OspC type B and L strains in human isolates from Slovenia and absence of OspC type A and K strains found in other regions of Europe suggests region-specific diversification of strains. It will be useful to determine whether there are regional variations in Lyme borreliosis in Europe and Asia, where *B. afzelii* and *B. garinii* predominate. Greater knowledge of regional differences in infection might help clinicians in diagnosis and treatment specific for their region.

In conclusion, *B. burgdorferi* from Europe and the United States represent distinct genotypes that vary in inflammatory potential and clinical manifestations of Lyme borreliosis. Despite greater genetic discordance *Borrelia* species, clinical features of *B. burgdorferi* infection in Europe appear similar to those for *B. afzelii* or *B. garinii* infection, the most prevalent *Borrelia* species in Europe, indicating that strains within a particular regional environment, under similar evolutionary pressures, accrue similar characteristics as other strains that share the same ecologic niche.
